# Learning and CRF-Induced Indecision during Escape and Submission in Rainbow Trout during Socially Aggressive Interactions in the Stress-Alternatives Model

**DOI:** 10.3389/fnins.2017.00515

**Published:** 2017-09-15

**Authors:** Tangi R. Summers, Torrie L. Summers, Russ E. Carpenter, Justin P. Smith, Samuel L. Young, Brandon Meyerink, T. Zachary Orr, David H. Arendt, Cliff H. Summers

**Affiliations:** ^1^Department of Biology, University of South Dakota Vermillion, SD, United States; ^2^Neuroscience Group, Division of Basic Biomedical Sciences, Sanford School of Medicine, University of South Dakota Vermillion, SD, United States; ^3^Veterans Affairs Research Service, Sioux Falls VA Health Care System Sioux Falls, SD, United States; ^4^Program in Writing and Rhetoric, Stanford University Stanford, CA, United States; ^5^Institute of Possibility Sioux Falls, SD, United States; ^6^Data Analytics, Sanford Health Sioux Falls, SD, United States; ^7^Alumend Sioux Falls, SD, United States; ^8^Children's Hospital Colorado—Research Institute Aurora, CO, United States

**Keywords:** corticotropin-releasing factor, social aggression, fear conditioning, Stress-Alternatives Model, SAM, retreat, explore, snap-shake

## Abstract

Socially stressful environments induce a phenotypic dichotomy of coping measures for populations in response to a dominant aggressor and given a route of egress. This submission- (Stay) or escape-oriented (Escape) dichotomy represents individual decision-making under the stressful influence of hostile social environments. We utilized the Stress-Alternatives Model (SAM) to explore behavioral factors which might predict behavioral phenotype in rainbow trout. The SAM is a compartmentalized tank, with smaller and larger trout separated by an opaque divider until social interaction, and another divider occluding a safety zone, accessible by way of an escape route only large enough for the smaller fish. We hypothesized that distinctive behavioral responses during the first social interaction would indicate a predisposition for one of the behavioral phenotypes in the subsequent interactions. Surprisingly, increased amount or intensity of aggression received had no significant effect on promoting escape in test fish. In fact, during the first day of interaction, fish that turned toward their larger opponent during attack eventually learned to escape. Escaping fish also learn to monitor the patrolling behavior of aggressors, and eventually escape primarily when they are not being observed. Escape *per se*, was also predicted in trout exhibiting increased movements directed toward the escape route. By contrast, fish that consistently remained in the tank with the aggressor (Stay) showed significantly higher frequency of swimming in subordinate positions, at the top or the bottom of the water column, as well as sitting at the bottom. In addition, a corticotropin-releasing factor (CRF)-induced behavior, snap-shake, was also displayed in untreated fish during aggressive social interaction, and blocked by a CRF_1_ receptor antagonist. Especially prevalent among the Stay phenotype, snap-shake indicates indecision regarding escape-related behaviors. Snap-shake was also exhibited by fish of the Escape phenotype, showing a positive correlation with latency to escape. These results demonstrate adaptive responses to stress that reflect evolutionarily conserved stress neurocircuitry which may translate to psychological disorders and decision-making across vertebrate taxa.

## Introduction

Adaptive behavioral responses to environmental stressors involve specific neurocircuitry-associated learning and conditioning, in all vertebrates, including rainbow trout (Carpenter and Summers, 2009). This type of learning and circuit activation is potently evoked by socially stressful stimuli such as territorial competitors, which dynamically modulate future behavior (Carpenter et al., 2009a). Fearful memories of a bigger and stronger competitor include important spatial and social information such as territorial boundaries, food, mates, opponents, and rank recognition that add salience to the fear memories of the aggressor (Johnsson, 1997; Forster et al., 2005; Korzan et al., 2006, 2007).

Ecologically relevant fear learning and memory formation drove development of an experimental model that takes specific contextual and social significance into account (Carpenter and Summers, 2009; Arendt et al., 2012; Smith et al., 2014, 2016; Robertson et al., 2015). The Stress-Alternatives Model (SAM) is designed to utilize stimuli that evoke social fear, and as such are often unpredictable, not habituated, and therefore result in significant stress (Summers et al., 2005). Social stressors appear to have the greatest salience and translatability for human psychological disorders (Haller and Alicki, 2012; Haller and Freund, 2013; Haller et al., 2013), perhaps because they have been judged to be the most potent stressors, even for dominant individuals that win aggressive interactions (Koolhaas et al., 1997).

Natural and domesticated populations of a wide variety of vertebrates appear to cope with stressful situations with a simple dichotomy of heritable strategies; either proactively or reactively (Benus et al., 1991; Koolhaas et al., 1999). Individuals with a proactive phenotype are characterized by behaviorally active coping, high aggression, low hypothalamus-pituitary-interrenal/adrenal (HPI/A) axis responsiveness, but high sympathetic reactivity. Reactive individuals exhibit passive coping, low aggression, elevated HPI/A responsiveness, and limited sympathetic reactivity. Behavioral and physiological components of coping styles exhibit a moderate to high degree of heritability (Driscoll et al., 1998; Ellenbroek and Cools, 2002; de Boer et al., 2003; Veenema et al., 2003), including in rainbow trout (*Oncorhynchus mykiss*) (Pottinger and Carrick, 1999). Social interactions in the SAM arena are generated by four (rodents) or seven (trout) days of interaction between a large novel aggressor and a smaller test animal, which produces a divergence of behavioral phenotypes, one actively coping with receipt of vigorous aggression through escaping, and a passive coping response in which the test individual remains submissively. The SAM experimental apparatus has been applied to experiments on trout, rats, hamsters, and mice (Robertson et al., 2015), and consists of an open field with one (trout) or two (rodents) escape routes only large enough for the smaller test individual. Consistent results for trout, rats, and mice suggest that escaping and submissive phenotypes are evenly distributed in populations, but also that the expression of active escape and passive submission constitute a decision-making process that is under the influence of neural stress circuitry and responses (Carpenter and Summers, 2009; Carpenter et al., 2009a; Smith et al., 2014, 2016; Robertson et al., 2015). This decision-making process is also subject to pharmacological manipulation resulting in reversal of choice.

Classical conditioning to aversive events in rainbow trout (Moreira et al., 2004), enhances our understanding of underlying neural circuitry producing dichotomous submissive and escaping phenotypes (Carpenter and Summers, 2009). In the SAM, social stress (aggression from a novel larger individual) when paired with a conditioned stimulus (CS, sound) produces a significant learned physiological response: Increased plasma cortisol to the CS alone (Carpenter and Summers, 2009). This Pavlovian response is only measured in submissive animals that do not escape, and whereas in the mammalian version of the SAM there is a clearly associated behavioral conditioned response (freezing), it wasn't obvious if there was a behavioral conditioned response (CR) that accompanied the physiological CR in trout (Carpenter and Summers, 2009; Smith et al., 2014; Robertson et al., 2015). We postulated that some behavioral responses, seen during the first day of SAM social interaction, would identify the development of submissive and escaping behavioral phenotypes, even before it was clear that those phenotypes existed. The purpose of this research, identifying predictive behavioral indicators and the potential neural plasticity and learning that accompany decision-making and the development of more stable stress-related behavioral phenotypes could provide tremendously practical information. In many vertebrate populations, understanding behaviors that predict susceptibility to stress, coincident with neuroplastic changes that result in heightened or reduced stress responsiveness, may help identify at-risk populations for behavioral inhibition, and humans vulnerable to anxiety, depression, and PTSD. Our recently developed SAM concept and apparatus was designed to probe the highly evolutionarily conserved decision-making that occurs during social stress and produces alternatives in behavior, and therefore reveals behavioral and neural indicators (Smith et al., 2014, 2016; Robertson et al., 2015, 2017).

In the SAM, provision of a route for egress creates the opportunity for a smaller test fish to learn to escape from an aggressive interaction that it cannot win; some individuals from the same population use this escape hole. It was not evident in previous experiments, what specifically drives escape or submission. In mice not faced with an aggressor, anxiety related to the open field (OF) aspect of the SAM arena stimulates withdrawal from the OF (Robertson et al., 2015; Smith et al., 2016), although trout alone in the SAM do not similarly use the escape route (Carpenter and Summers, 2009). In both mice and trout, test animals that remain submissively with a large aggressor will change phenotype and begin to use the escape hole when treated with an anxiolytic drug, the corticotropin-releasing factor type 1 (CRF_1_) receptor antagonist, antalarmin. In mice, the anxiogenic drug yohimbine, a noradrenergic α_2_ receptor antagonist, blocks escape in most animals, which then remain submissively in the OF with an aggressor. These results suggest that multiple integrative stress neurocircuitries play a role in determining behavioral phenotype, and that these phenotypes are flexible (Smith et al., 2016). They also suggest that the test animals invoke decision-making neurocircuitry, which overlaps with the stress neural pathways, to choose the phenotype most suited to its emotional state, both of which are reversible (Smith et al., 2014).

As CRF clearly plays a role in trout social behavior (Carpenter et al., 2009a; Backström et al., 2011a,b; Backström and Winberg, 2013), and because previous results suggest that CRF is associated with anxiety and indecision (Carpenter et al., 2007, 2009a; Backström et al., 2011a), we hypothesized that this anxiogenic neuropeptide generates stereotypic behavior as it does in other vertebrates. In addition, we hypothesized that CRF-induced anxiety-driven behavior would result in hesitation during the decision-making process for escaping and submissive phenotypes. During this investigation we discovered a new behavior, linked directly to CRF, which we named Snap-Shake. We hypothesized that this behavior would increase in frequency during the process of choosing whether or not to use the escape hole, and because it is stimulated by a known anxiogenic neuropeptide and is associated with anxiety-causing behavioral decision, we hypothesized that snap-shake would act as a marker of indecision. Furthermore, we hypothesized that in the face of aggression-induced anxiety and indecision the efficiency of escape behavior would be a product of social learning. Based on information from all of the SAM models, we hypothesized that aggression amount or intensity does not specifically stimulate escape from the social interaction, but that other behavioral responses early in the social interaction would identify which animals would rely on submission, and which would choose egress to cope with social aggression.

## Materials and methods

### Subjects and housing

Social interactions using the fish version of the SAM arena were conducted at the Gavins Point National Fish Hatchery in Yankton, South Dakota. Rainbow trout (*O. mykiss*; raised from broodstock eggs provided by Ennis National Fish Hatchery in Montana) were reared in 50 foot long cement raceways under natural light conditions. The raceways were continuously supplied with aerated well water at 12°C. Fish were fed daily (Nelson's Silver Cup, Murray, UT, USA) at a rate of 1% body weight per day throughout the entire experiment. Large (350–570 g) adult hatchery brood-stock (*N* = 21) were used as the aggressive social stimulus (US) in this experiment. These fish were housed separately prior to experimentation, rotated and rested throughout the experiment to insure a high level of aggression toward the test fish. All experiments were conducted in a manner that minimized suffering and the number of animals used, in accordance with the *National Institutes of Health* Guide for the Care and Use of Laboratory Animals (NIH Publications No. 80–23), under a protocol approved by University of South Dakota IACUC.

### Acclimation

Prior to training, small, juvenile (79–209 g) test fish (*N* = 80) were netted out of the group raceway and allowed to acclimate for 10 days in individual compartments of 75 gallon glass tanks with a flow-through water system and aeration. Each tank was individually lit and separated into four equal-sized compartments by the insertion of three opaque Plexiglas barriers. These UV lights were on at 6 a.m. off at 6 p.m. Fish were exposed to only ¼ of the tank during acclimation. The time for acclimation was chosen to stimulate territorial association with each compartment, while barriers inhibited social interaction between fish.

### Experimental design

**Figure 1:**
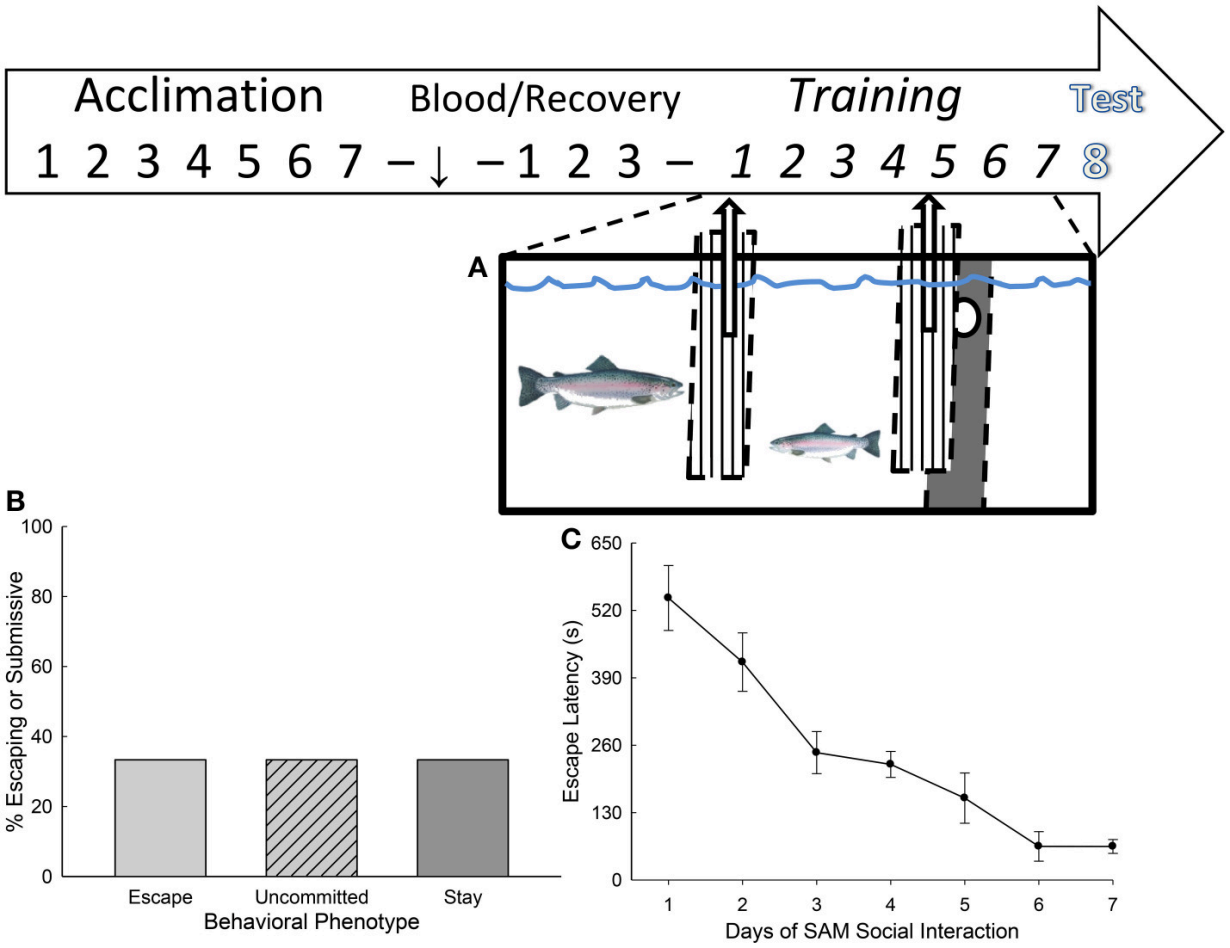
Stress-Alternatives Model aquatic arena and experimental design. **(A)** The investigation design time line included a week of acclimation to experimental tanks, followed by pretest blood sample 3 days of recovery, and then training. Social interactions with a novel large aggressor (US) occurred daily in the SAM (training) following CS presentation (water off) over the next week, with testing on the following day. Fifteen minutes after the initiation of testing terminal blood and brain samples were collected for analysis. **(B)** The percentage of trout that display the Escape (light gray bar, *N* = 10) or Stay (dark gray *N* = 10) phenotypes reliably, with the remaining animals uncommitted to either (light gray hatched). Uncommitted trout displayed escape during some social interactions, and Stay during others. **(C)** The learning curve for escape. Mean (± SEM) latency to escape for each day of training (1–7).

Social Stress in the SAM arena (Figure 1) typically produces a reliable dichotomy of behavior (Carpenter and Summers, 2009; Smith et al., 2014, 2016; Robertson et al., 2015) in small test fish (*N* = 30) that received a trace signal 15 s before social interaction with the possibility of escape through a hole (accessible by all smaller fish). Fish choose, on their own, whether to escape (Escape) or remain submissively (Stay). Groups were designated *a priori* based on previous results, but were assembled only after behaving, to include: (1) trout that escape social interaction with a larger aggressor (Escape), and (2) those that remain submissively with the novel larger fish (Stay). During social interaction, all fish were exposed to ½ of the tank by divider removal. While all fish had the opportunity to escape using the hole, only those that did so had the possibility of exploring a 3rd compartment of the tank accessible only by the hole; these fish had no pre-exposure to the hole.

Another cohort of 32 fish was either untreated (*N* = 8), or anesthetized (500 mg/l methane tricane sulfonate) and injected icv directly into the 3rd ventricle (Clements et al., 2002), by lowering a 26 gauge needle into the brain postorbitally along the midline to a depth of 7 mm (verified by methylene blue staining with >95% accuracy) with one of the following: 0 (injection control, *N* = 8; 2 μl of aCSF only: 0.2% NaHCO3 in 0.6% NaCl), 500 (*N* = 8), or 2,000 (*N* = 8) ng CRF (ovine corticotropin releasing factor; Sigma, St. Louis, MO, USA) (Clements et al., 2002) in 2 μl of artificial cerebrospinal fluid (aCSF) to examine the relationship between CRF and snap-shake behavior. Snap-shake is a behavior that combines biting motions, opercula flaring, and head shaking (Abbott and Dill, 1985; Carpenter et al., 2007; Backström et al., 2011a). In video analysis of social behavior with no pharmacological treatment, we observed snap-shake behavior and recognized it as the behavior observed following CRF treatment. An additional group of trout (*N* = 18) were observed through the complete set of SAM social interactions and treated with the CRF_1_ receptor antagonist antalarmin to determine whether endogenous CRF plays a role in Snap-Shake behavior in response to anxious indecision related to escape. For these trout, on day 4 of SAM social interactions, individuals from both escaping (*N* = 10) and non-escaping (*N* = 8) groups were orally administered the brain-permeant CRF_1_ receptor antagonist antalarmin (2 mg/Kg) in food, and assessed for snap-shake behavior.

### Social interaction

Following 10 days, which includes 7 days of acclimation, initial blood sampling, and a 3 day recovery period, fish were exposed to 7 days of social interaction with one session per day. Beginning with Day 1, water inflow to the tank was turned off (CS), and 15 s later, the barrier separating the large (US) and small fish was removed, along with the barrier that covered the escape hole (~3.8 cm dia.), making a neutral chamber available to the test fish. Fish were allowed to interact for 15 min, and latency to first attack, aggressive-, submissive-, and escape-related (retreat/explore) behaviors plus escape time (if applicable) were recorded. If the test fish did not escape within 15 min, the fish were separated, barriers re-inserted, water inflow was turned back on and the interaction was over. If the test fish did escape, the water was turned back on, the big fish moved back to his chamber, and the small fish was allowed to remain in the empty compartment for 5 min before being ushered back into the home chamber (by removing the divider). Pairings of CS:US occurred once daily over 7 days. Each test fish interacted with a novel large fish every day. Following 7 days of social interaction, on experimental day 8, fish were presented with the CS only (water off and barriers removed) for 15 min. No large fish was present. Behavior was recorded for 15 min. Immediately following this observation, test fish tissues were rapidly collected for analysis. Previous experiments also included aggression controls, in which pairs contested without access to an escape hole, as well as hole-only controls in which test fish were not paired with an aggressor, but did have access to the escape hole (Carpenter and Summers, 2009). In the absence of an aggressor, either during the 7 days of trials or on the 8th test day, test fish never used the hole to escape, in contrast to similar controls in mice that always escaped (Carpenter and Summers, 2009; Smith et al., 2014, 2016).

### Behavioral analysis

During interactions, observers (unaware of treatment group) recorded time of first attack and intensity of aggression by the larger fish, snap-shake anxious behavior, and position in water column of test fish, as well as aggressive, submissive, and retreat-explore (escape-related) behaviors of the test fish. Time of escape (if any, for those that use the hole) was also recorded. Latency to escape is calculated from the time of first attack (bite, attempt to bite, or chase with attempt to bite). The anxious behavior that we refer to as snap-shake is an integrated combination of biting motions, opercula flaring, and head shaking that occur together (Abbott and Dill, 1985; Carpenter et al., 2007; Backström et al., 2011a). For the comparison of the number of snap-shakes per social bout with latency to escape, we had to take into consideration that the latency diminishes over the duration (7 days) of the experiment. Therefore, each latency to escape must be adjusted for the learning that accrues with each trial resulting in diminishing time to escape, for the comparisons of snap-shake to be meaningful. We used the simple adjustment of dividing each daily latency to escape, by the average escape latency for all fish on that day. The result is a ratio of individual latency to average group latency, such that scores above one represent indecisive fish, with slower than average escape latency, and scores less than one representing fish eager to escape, completing the task faster than average. For intensity of attacks received in a social interaction, we used the number of attacks occurring in any 3 s period during the total 15 min interaction. Intensity of aggression was measured for 3 s bins according to these levels: 1 attack during any 3 s period = low intensity, 2 attacks within 3 s = medium intensity, and 3 or more attacks within this time frame = high intensity aggression. Behavior was recorded on a Canon Vixia HG20 High Definition Camcorder. Video recordings were transformed to MPEG files and viewed on WINDVD Intervideo software.

### Statistical analysis

For all comparisons between groups (Escape vs. Stay), including analyses for intensity of attacks received, percent of snap-shake interactions, total submissive behavior exhibited over time, retreating/exploring over time, aggression over time, turning toward the attacker, movement directed at the escape hole, swimming at the bottom of the tank, or sitting at the bottom, Student's *T*-tests were utilized. Analysis of percent time spent in submissive, retreat/exploring, and aggression, across all three types of behavior were compared for each group (Escape or Stay) using one-way ANOVA, including the analysis relative to behavior that preceded snap-shake. Linear regression analysis compared relationships between latency/escape learning curve and snap-shake performance and for number of escapes vs. accumulated social interaction in which the aggressor was not watching. For observed escapes, a curvilinear regression fit the data more precisely, and was therefore used. For all analyses, each animal provided only a singular datum. The data have been tested for the five assumptions of parametric statistics and transformed (log) when necessary. The data are analyzed both non-parametrically and using the parametric statistics previously mentioned, and for multiple comparisons using the Holm-Sidak method; when the statistical analyses match, as they do for the data reported herein, we report the parametric results (using tests listed above) without α adjustment to avoid increased Type II error (Rothman, 1990; Perneger, 1998; Feise, 2002; Jennions and Moller, 2003; Moran, 2003; Nakagawa, 2004). Significant effects between groups for one-way analyses were examined with Student–Newman–Keuls *post-hoc* analyses (to minimize Type I error) and Duncan's Multiple Range Test (to minimize Type II error).

## Results

### Expression of behavioral phenotype

As in previous experiments, social interaction with a significantly larger individual and the opportunity to leave the social arena produced two kinds of response: Escape and Stay. These behavioral phenotypes were equally distributed in the population (*N* = 10 for each group), with a third type of response not previously reported (Figure 1B). Escaping fish do so more rapidly each day (Figure 1C), such that the daily means for latency to escape over time describe a learning curve for the population. Trout that Escape use the hole to leave the social arena in the aquarium five to seven times over the week of training, whereas those that Stay submissively with the larger aggressor may use the hole once or twice during the week, but more typically do not do so at all. Interestingly, there were also 10 fish that used the escape hole three or four times each during the week of social interactions, choosing escape on some days, but not on others. The results suggest that an additional group, not previously reported, may require more time before adhering to a specific behavioral phenotype.

### Effects of aggression on choice of behavioral phenotype

**Figure 2:**
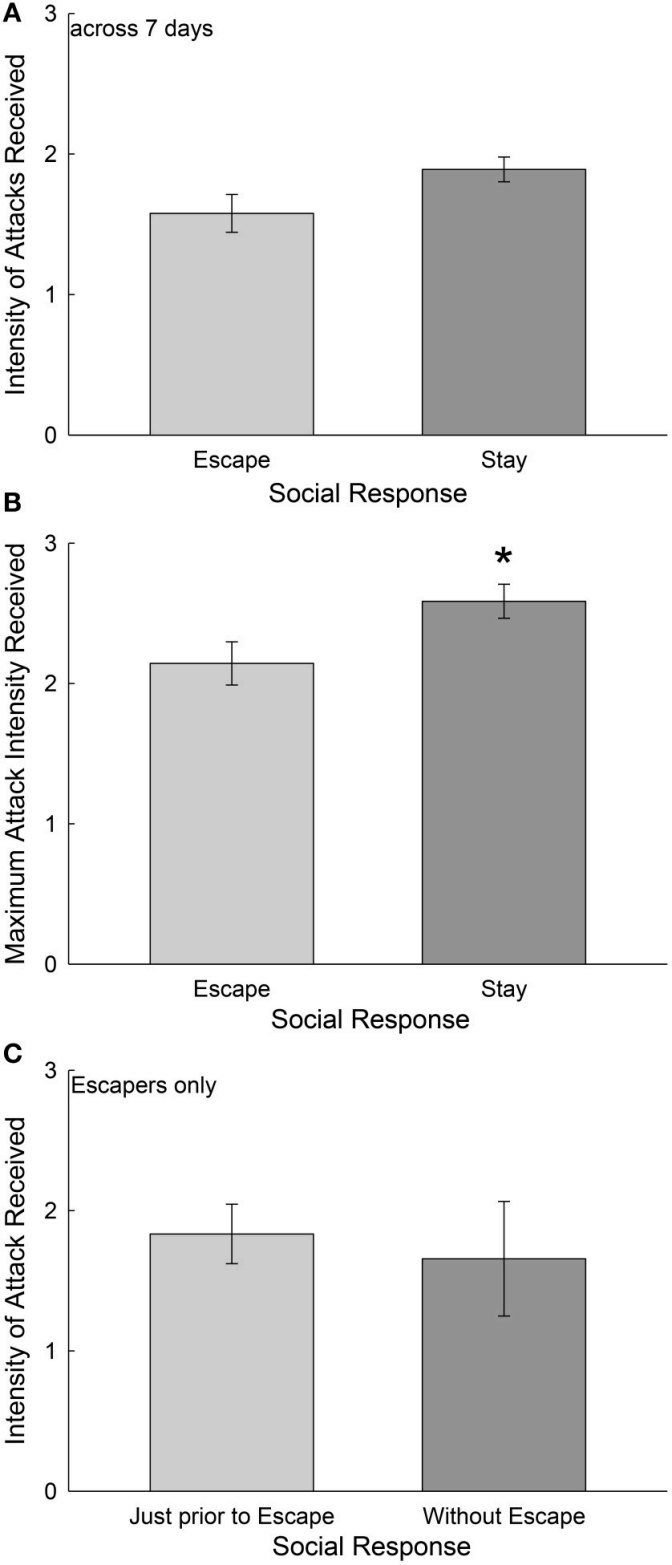
Escape behavior is *not* precipitated by aggression. **(A)** Mean (± SEM, Student's *T*-tests) intensity of attacks received by the test animal, where intensity of attacks is determined by a scale of aggressive events in which one, two, or three (or more) attacks occur within any 3 s period over the entire duration of social interaction. Daily attack intensity means on this scale are averaged for each individual over 7 days of social interaction to produce one data point. The mean for these data are represented for animals that Escape from (light gray bar, *N* = 10) and for those that Stay with (dark gray bar, *N* = 10) a daily novel larger aggressor. **(B)** Mean (± SEM) maximum daily attack intensity, using the same scale. Trout that remain submissively (Stay) receive significantly (^*^) higher maximum intensity attacks than do escaping fish. **(C)** Mean (± SEM) attack intensity 30 s prior to escape (light gray bar) and averaged across the entire 15 min social interaction when animals that usually escape do not utilize the escape route.

To examine the role of aggression in determining whether test fish escaped from the larger aggressor (Escape) or remained submissively (Stay) we examined attack intensity in three ways: (1) Average attack intensity received over each of 7 days of social interaction in the SAM, (2) the maximum attack intensity each day, averaged over 7 days, for Escape and Stay groups. Finally, we examined in the Escape group only, (3) the maximum intensity of attack received each day just prior (30 s) to escape. On the rare occasions when Escape group individuals did not escape, we used the maximum daily attack intensity (2) for comparison. For intensity of attacks received in a social interaction, we used the number of attacks occurring in any 3 s period as a gradient of intensity of attack that ranged from one (low intensity) to three (high intensity). When the intensity of attacks is averaged each day, and then over all 7 days, there is no broadly significant effect of aggression compelling escape behavior; and by contrast there is a trend for the Stay group [*t*_(18)_ = 1.945, *p* < 0.068] to have received more attacks than the Escape group (Figure 2A). Similarly, when we examined the maximum average attack intensity on a given day (the greatest intensity, 1–3, then averaged over 7 days), trout remaining submissively with the larger aggressor (Stay) received significantly greater [*t*_(18)_ = 2.26, *p* < 0.037] intensity of attack than escaping animals (Figure 2B). Finally, considering the Escape group alone, when we compared the maximum attack intensity received in the final 30 s prior to escaping through the hole to the average maximum attack intensity received when on the rare occasion they did not escape, there was no significant difference [*t*_(18)_ = 0.38, *p* > 0.71; Figure 2C].

### Snap-shake behavior relates to choice anxiety

**Figure 3:**
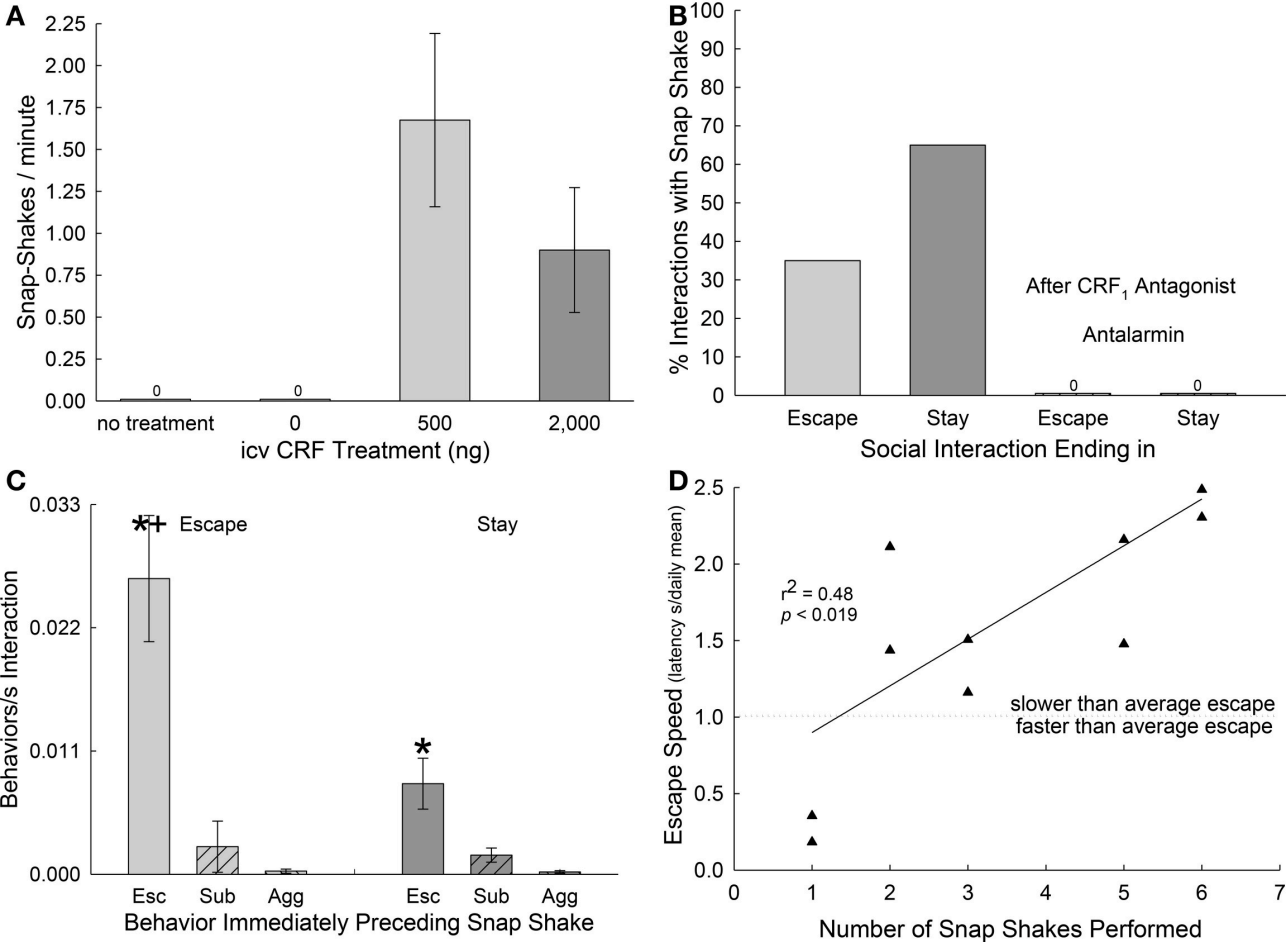
Snap-shake behavior is induced by CRF and epitomizes indecision regarding escape. **(A)** Mean (± SEM) number of snap-shake behaviors expressed per minute without treatment (*N* = 8), following icv injections of aCSF (= 0 ng CRF, *N* = 8), or corticotropin-releasing factor (CRF; *N* = 8 each for 500 or 2,000 ng). **(B)** Percentage of social interactions that include snap-shake behavior in Escape (light gray bars, *N* = 10) and Stay (dark gray bars, *N* = 10) phenotypes. **(C)** Mean (± SEM) number of snap-shake behaviors per second of interaction immediately following retreat/explore- (Esc; bars without hatching), submission- (Sub; bars with left hatching), and aggression-related (Agg; bars with right hatching [hatching not visible]) behaviors in Escape (light gray bars) and Stay (dark gray bars) phenotypes. Escape behavior precedes snap-shake significantly (^*^one-way ANOVA) more often than other behaviors in both phenotypes (^+^T-test, Escape significantly greater than Stay; *N* = 10 each). **(D)** Positive linear regression (*r*^2^ = 0.48, *P* < 0.019, *N* = 10) between escape speed, normalized for daily changes in the escape learning curve (see Figure 1C), and number of snap-shakes performed. The dotted line represents the demarcation between slower (above) and faster (below) than average escape, and results from the normalization formula: individual latency to escape in seconds/group mean escape latency (s) for that day.

We examined the role of uncertainty to the establishment of a firm behavioral phenotype, by using the snap-shake behavior as a marker of indecision. We first set out to determine whether snap-shake was related to CRF levels in the brain by injecting this neuropeptide into the cerebral ventricles (icv) of isolated fish (no aggressor present). While trout injected icv with aCSF never displayed snap-shake, which was also true for untreated fish, 75% of those injected with 500 or 2,000 ng CRF exhibited the behavior. The rate of snap-shakes/minute in icv CRF injected fish ranged from 0.9 to 1.68 (Figure 3A). Having established the role of CRF on stress, anxiety (Carpenter et al., 2009a; Backström et al., 2011a) and performance of snap-shake behavior, we next examined with which behavioral phenotype snap-shake was most often associated. While escaping trout exhibited snap-shake in 35% of the interactions of this group (Escape), animals that remained submissively with an aggressor (Stay) displayed the behavior nearly twice as often (65%); while snap-shake in both phenotypes was inhibited completely by the CRF_1_ antagonist antalarmin (Figure 3B). However, in both Escape and Stay phenotypes, the category of behaviors that immediately precede snap-shake is escape-related (retreat/explore) behavior. Escape-related behavior was significantly more likely to precede snap-shake in the Escape group than submissive behavior or aggressive behavior [*F*_(2, 42)_ = 9.2, *p* < 0.001; Figure 3C, left hand bars]. Even in submissive fish (Stay group), behaviors related to escape (such as noticing the escape route or pausing in front of the hole), but not using the escape hole *per se*, preceded snap-shake behavior significantly more often than submission or aggression [*F*_(2, 39)_ = 9.4, *p* < 0.001; Figure 3C, right hand bars]. As submissive fish (Stay) react with snap-shake behavior more frequently, but do so primarily in response to behavior associated with the escape hole, we hypothesized that snap-shake behavior reflects the anxiety of decision-making related to escape. For that reason, we examined the relationship between the manifestation of rapid escape (expressed as the individual escape latency divided by the group average for that day) and the number of snap-shakes performed. As a significant positive regression [*F*_(1, 9)_ = 8.22, *p* < 0.0186, *r*^2^ = 0.48; Figure 3D] indicates that as the tendency to resolve the execution of escape is delayed the more snap-shakes are performed. Most of the escaping fish that show snap-shake have slower than average escape latencies (Figure 3D, above the dotted line). Those fish that put off escaping display more snap-shake behaviors, expressing measurable indecision related to making use of the hole, and indicating that they skew the shape of the escape learning curve toward slower responses for that fish during that interaction.

### Social environment shapes escape behavior

**Figure 4:**
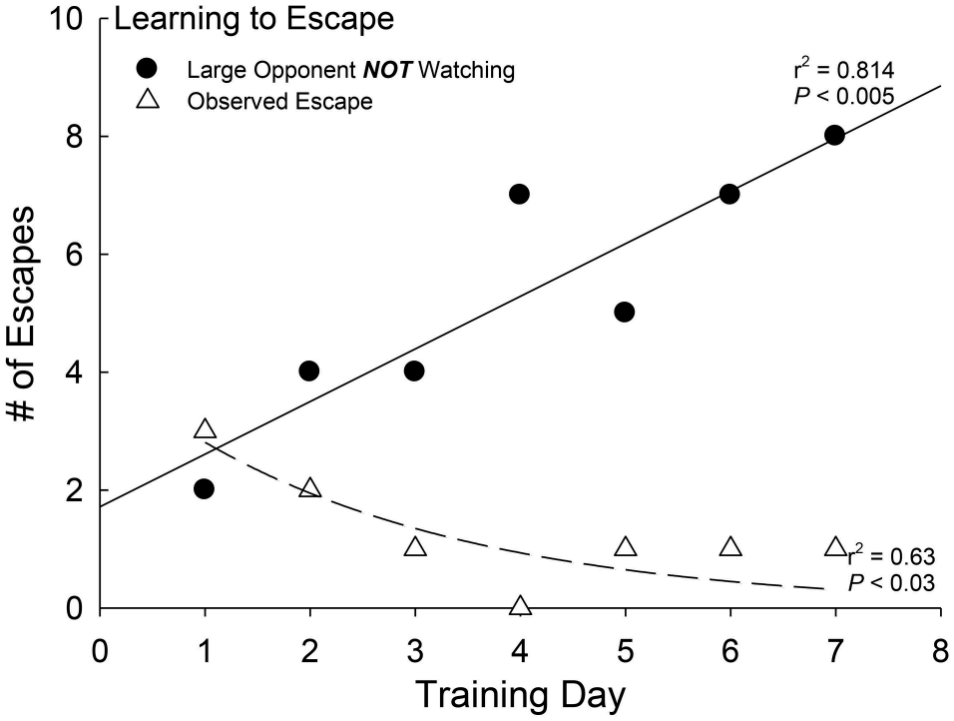
Social learning precedes efficient escape. Positive linear regression between the frequency of unobserved (larger aggressor is incapable of viewing the test fish (*N* = 10) and the escape route) escapes and days of social interaction experience (black dots, solid regression line; *r*^2^ = 0.814, *P* < 0.005). Negative curvilinear regression between the frequency of observed escapes (by larger patrolling aggressor) and number of days of experience (each with a novel aggressor) of social interaction (white triangles, dashed regression line; *r*^2^ = 0.64, *P* < 0.03).

As a social environment produces similar anxious behavior as CRF and the HPI/A endocrine cascade (Carpenter et al., 2007, 2009a; Backström and Winberg, 2013), we examined the relationship between patrolling behavior by the large aggressor and escape behavior in test trout. First we examined whether unobserved escape would increase as the fish became more proficient at leaving the social interaction arena (Carpenter and Summers, 2009). During the first day of social interaction, the number of escapes performed when the larger aggressor fish was not able to observe the smaller test fish use the escape route were few, but that number grew steadily and significantly over time [*F*_(1, 5)_ = 21.85, *p* < 0.005, *r*^2^ = 0.814; Figure 4, black dots]. Similarly, but in the opposite direction, observed escapes declined as the number of days of social interaction progressed [*F*_(1, 5)_ = 8.33, *p* < 0.034, *r*^2^ = 0.63; Figure 4, white triangles]. Careful video analysis revealed that the smaller (test) fish were learning to escape when the larger aggressor was turned away and not looking.

### Certain behaviors predict escaping and submissive phenotypes

**Figure 5:**
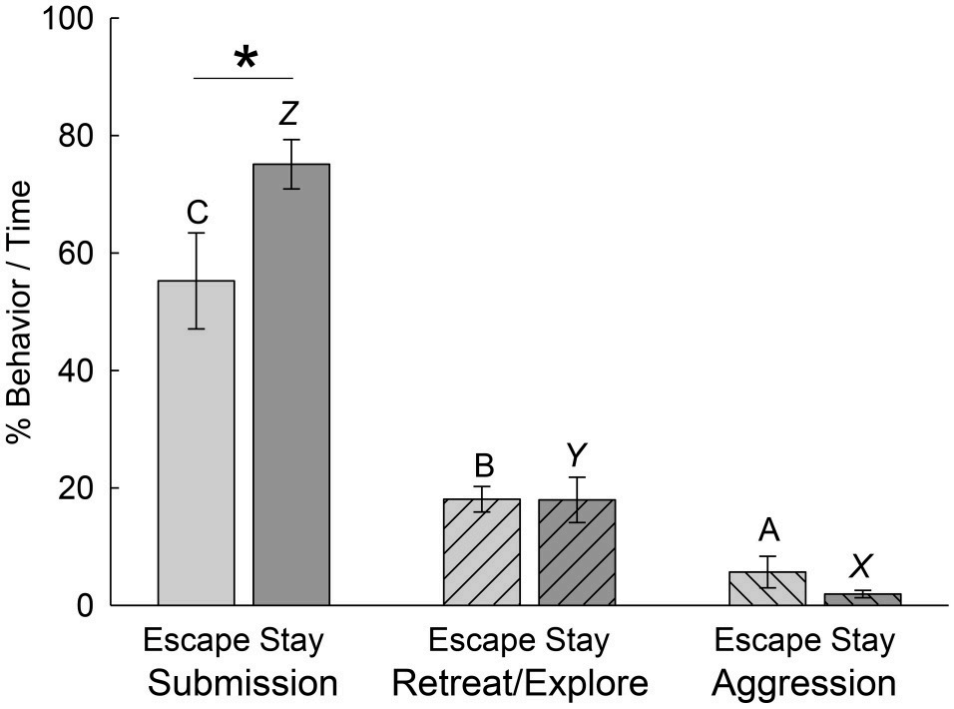
Submissive behavior is more prevalent in Escape and Stay phenotypes during the first day of social interaction. Mean (± SEM) percentage of submission- (bars without hatching), retreat/explore- (left hatching), and aggression-related (right hatching) behavior per second of the first social interaction in Escape (light gray bars, *N* = 10) and Stay (dark gray bars, *N* = 10) phenotypes. For both phenotypes submission-related behaviors occur significantly (bars with differing letters are significantly different, one-way ANOVA; i.e., C vs. A or B for the Escape phenotype, or *Z* vs. *X* or *Y* in the Stay phenotype) more frequently than other behaviors; and retreat/explore-related behaviors occur significantly more frequently than aggression-related behaviors (Escape: B vs. A; Stay: *Y* vs. *X*). Animals expressing the Stay phenotype show significantly (^*^P < 0.05) more submission than those expressing the escape phenotype.

Although there was no clear behavioral Pavlovian conditioned response in trout that was coincident with the classically conditioned cortisol response (Carpenter and Summers, 2009), we examined a series of behaviors during the first day of social interaction in each phenotype to determine if any were predictive of the developing Escape and Stay phenotypes as they became established. We first compared all first day submission, retreat/explore (escape-related behavior), and aggression behaviors that accumulated over the first day of social interaction for each phenotype. While we didn't expect this gross examination to produce definitive distinguishing characteristics, the results were interesting nonetheless. For both groups of animals that will eventually be sorted into Escape [*F*_(2, 27)_ = 25.38, *p* < 0.001] and Stay [*F*_(2, 27)_ = 135.04, *p* < 0.001] groups after 7 days of social interaction, when limiting the analysis to behaviors on only the first day alone, submissive behaviors were by far the most prevalent, and occurred significantly more often than retreating, exploring, or aggressive behaviors (Figure 5). For both phenotypes, retreating/exploring behaviors (related to escape) were more prevalent than aggressive behavior. Since the opponent was three times larger than the test fish, it wasn't surprising that aggression-related behaviors were very uncommon. Comparing within these three categories by phenotype, submissive behaviors were significantly [*t*_(18)_ = 2.19, *p* < 0.042] more common for the Stay than the Escape group. In contrast, retreating/exploring [*t*_(18)_ = 0.016, *p* > 0.988] and aggressive [*t*_(18)_ = 1.361, *p* > 0.19] behaviors were similar between the two phenotypes.

**Figure 6:**
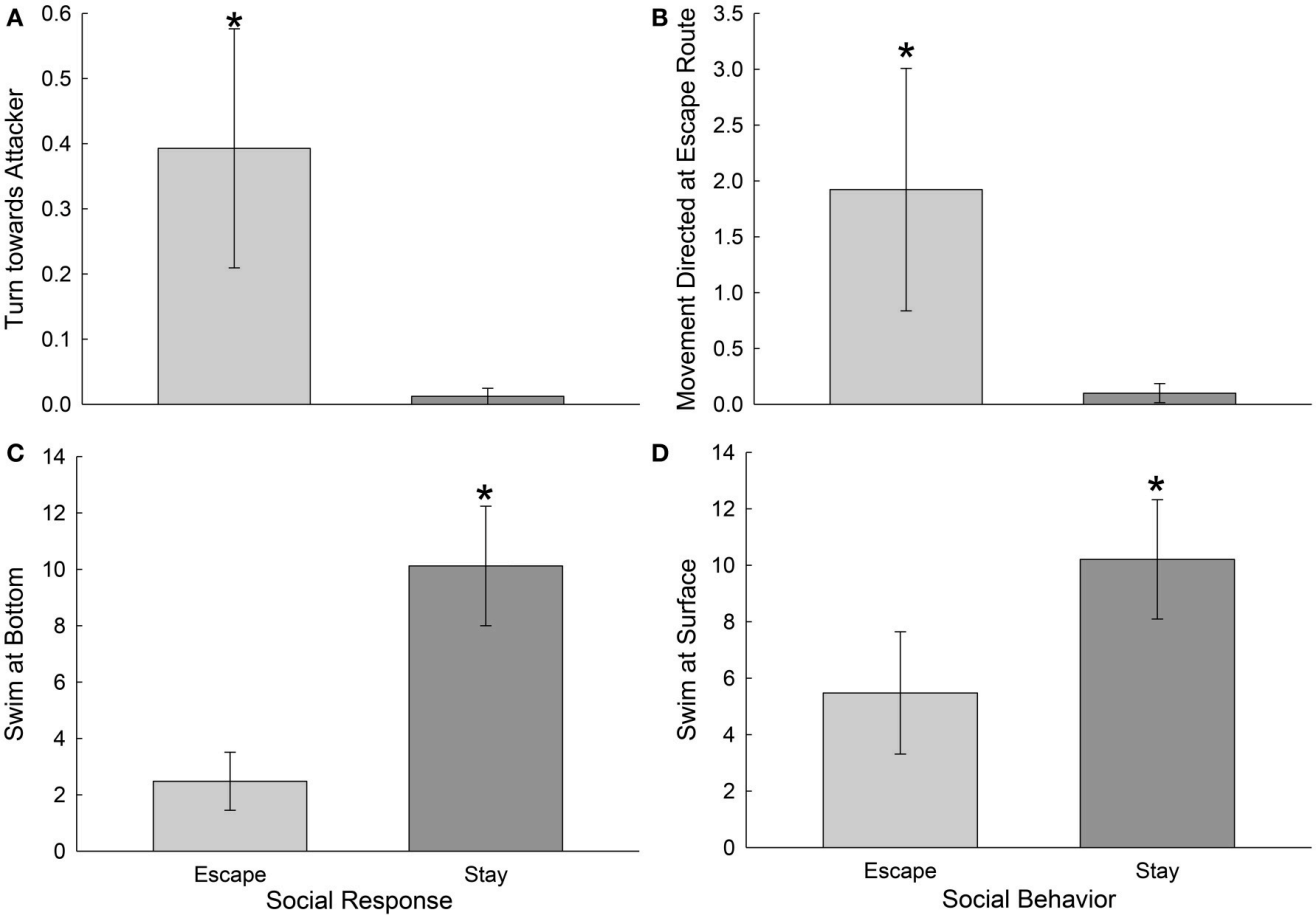
Escape and Stay phenotypes are predicted by specific distinctive behaviors prior to establishment of the phenotype. **(A**) Mean (± SEM) number of turns toward the attacker per second of interaction, are significantly greater (^*^Student's *T*-tests) in Escape fish (light gray bars, *N* = 10) that in those that Stay with the larger aggressor (dark gray bars, *N* = 10). **(B)** Mean (± SEM) number of movements directed at the escape route per second of interaction are significantly greater (^*^) in Escaping than Staying trout. **(C)** Mean (± SEM) occurrences of swimming at the bottom (or sitting at the bottom; data not shown) or **(D)** top of the tank per second of interaction are significantly greater (^*^) in trout that Stay, rather than Escape.

With more detailed inspection of specific behaviors during the first day of social interaction, it became clear that a few were clearly predictive for submissive and escaping phenotypes prior to their overt establishment. Fish that reliably escaped on most of the 7 days of social interaction (Escape group), were significantly more likely to turn toward their attacker on day one than were fish that reliably stayed with the novel aggressor on most of the 7 days of interaction [*t*_(18)_ = 2.1, *p* < 0.05; Figure 6A]. These fish also displayed more movement directed at the escape route on day one than submissive fish [*t*_(18)_ = 5.85, *p* < 0.001], before they actually escaped (Figure 6B). Submissive fish (Stay Group), on the other hand, were significantly more likely to swim at the bottom [*t*_(18)_ = 3.24, *p* < 0.005] or at the surface [*t*_(18)_ = 2.2, *p* < 0.05], and to sit at the bottom [*t*_(18)_ = 1.87, *p* < 0.05, data not shown] on the first day of social interaction, than fish that would eventually escape (Figures 6C,D).

## Discussion

Social interactions in the SAM apparatus produce dichotomous phenotypes that are not evident prior to SAM experiments (Smith et al., 2014) at least in mammals, and not driven uniquely by the amount or intensity of aggression in trout (Figure 2) and rodents (Prince et al., 2015). The decision-making process that produces strikingly different behavioral responses are shaped by central CRF and noradrenergic systems in fish (Carpenter et al., 2009b; Robertson et al., 2015) and mice (Smith et al., 2016). The central CRFergic response, while shaping anxiety (Carpenter et al., 2007, 2009a; Backström et al., 2011a; Backström and Winberg, 2013) and stress responses, also appears to produce stereotyped behavior in trout, snap-shake, which appears to represent the anxiety of indecision regarding using the unfamiliar escape route (Figure 3). While social experience teaches test fish to use information regarding their aggressor for Escape vs. Stay decisions (Figure 4), they already begin to show distinguishing behaviors on the first day of social interaction with an aggressor (Figures 5, 6), prior to clear establishment of the full behavioral phenotype.

We were surprised that amount or intensity of aggressive behavior did not have anything to do with whether animals chose to escape from the SAM arena, or to stay with the larger aggressor and continue to be exposed to attacks (Figure 2). However, this result in salmonid fish, is exactly mirrored by experimental results in mice from the rodent oval-open field SAM arena (Prince et al., 2015; Robertson et al., 2015). These results indicate that the most fearful animals are not driven from the interaction arena by aggression. Additionally, in both mice and trout, there are some individuals that do not develop consistent Escape or Stay phenotypes within the normal experimental duration of seven social interaction days for trout, or four for mice. We have chosen to limit our interpretation of the results to those that most of the daily outcomes match one phenotype, because analysis of reversible decision-making is challenging, and not complete. It appears however, that the variability does not derive from inconsistency in aggression received.

For those individuals that reliably Escape or Stay, the SAM concept provides a method to determine varying intensity of anxiety and stress (Smith et al., 2016). This gradient of anxiety and stress depends in part on actions of central CRF in both trout and mice, in which remaining submissively with the larger aggressor can be reversed in some individuals with an acute application of the CRF_1_ receptor antagonist antalarmin (Carpenter et al., 2009b; Robertson et al., 2015; Smith et al., 2016).

Interestingly, central CRF appears to play an important role in the decision-making process as well, apparently promoting indecision (Figure 3). Intraventricular injections of CRF promote stereotyped behavior in familiar environments in rodents (Cole and Koob, 1989; Matsuzaki et al., 1989; Holahan et al., 1997; Salak-Johnson et al., 2004) and fish (Carpenter et al., 2007; Lastein et al., 2008). In trout this behavior is a combination of head shaking, flaring opercula, and biting motions, that we call snap-shake (Abbott and Dill, 1985; Carpenter et al., 2007; Backström et al., 2011a). Although previous experiments reported CRF-induced snap-shake during aggression, it had never been observed in untreated fish. Here we report that snap-shake does occur naturally, and accompanies primarily escape-related behavior in trout that do not escape. Male and female test animals exhibit snap-shake behavior during interactions with both male and female aggressive opponents, but never without opponents (except with CRF treatment) or after escape. Snap-shake behavior is expressed during interactions that range from no aggression (rare) to the highest level of aggression (also rare), but occurs most often in bouts that reach strong levels of aggression. In 56 interactions with a larger fish and no escape possible (no hole present), snap shake behavior did occur, but much more rarely (11% compared to 60% when escape was possible). Snap-shakes are often seen near the escape hole, but sometimes it appears the fish is doing snap-shakes after actively avoiding the hole. In 150/249 interactions where snap-shake was observed when an escape route was present, the behavior preceding snap-shake was near the hole. Escaping trout also perform snap-shake, although less frequently, and when they do, it appears to correlate well with indecisiveness about escape itself, suggesting that it may be a reliable measure of anxiousness, and perhaps even central CRF levels, since the CRF_1_ receptor antagonist antalarmin effectively blocked snap-shake during the period during which anxious decision-making leads to escape or remaining submissively (Figure 3B). The number of snap-shake behaviors performed in escaping animals positively correlates with the escape speed (normalized for daily learning by: individual escape latency / group latency mean for that day) in these fish. The longer it takes them to use the escape hole, the more snap-shakes are performed (Figure 3D). In addition, most fish that exhibit snap-shake also have slower than average escape. These data, together with the reversibility of remaining submissively following the anxiolytic CRF_1_ antagonist treatment (Carpenter et al., 2009b; Smith et al., 2016), suggest that trout Staying with the large aggressor do so primarily because their central stress neurocircuitry is more reactive (Carpenter et al., 2009b; Robertson et al., 2015). This conclusion is corroborated by similar results from the SAM arena for mice (Robertson et al., 2015; Smith et al., 2016). Stress vulnerability is ameliorated by the act of escaping, which also depends on critical learning steps related to the social interaction (Carpenter and Summers, 2009). Interestingly, the steps critical for learning escape are accrued in reliably escaping fish during 7 days of interaction, submissive fish also learn how to escape during that time, but do not apply the knowledge (Carpenter et al., 2009b). The act of escape, of course, allows the animal to learn that the chamber to which they escape is a safe place, also leading to amelioration of anxiety.

When the smaller test fish escapes through the hole leading to a safe adjacent compartment, it does not happen efficiently unless it pays attention to the novel larger patrolling trout (Figure 4). During the first few days of social interaction most escape attempts occur under the watchful eye of the large aggressor. This larger fish patrols the tank relentlessly, and often rams or bites at the smaller fish attempting to leave. So with time, the smaller test fish learn to apply a strategy that relies on swimming to the escape hole when the large patrolling fish is not looking. Usually that means that the large fish is headed in the other direction, and is some distance from the escape route. As the days of social interaction progress, significantly more escapes occur when the larger trout cannot observe the smaller fish passing through the hole. Conversely, observed escapes diminish in frequency as the days of social interaction proceed. The results suggest that a significant amount of learning takes place during this decision-making process. We also hypothesized that during this decision-making process, in the first social interaction there would be clear behavioral differences that predict which phenotype an individual will adopt before the Escape or Stay choice has been reliably repeated, and translated into a specific and relatively stable social phenotype.

Our results should be interpreted within the scope of the SAM's capacity for generating and revealing stress, affective, and gene expression responses. The SAM reveals anxious behavioral responses that vary in intensity along a continuum or gradient (Robertson et al., 2015; Smith et al., 2016). As the intensity of anxious behavioral responses grows in mice, plasma corticosterone and gene expression of the anxiety responsive neuropeptide S (NPS) in the central amygdala (CeA) rise commensurately (Smith et al., 2016). The continuum of CeA NPS expression and corticosterone concentration is evident in Escape and Stay behavioral phenotypes, and while elevated in both groups, significantly greater in submissive (Stay) animals. Similar changes (some elevated and some diminished) are revealed following social interaction in the SAM coupled with fear conditioning (FC) on brain-derived neurotrophic factor (BDNF) and its receptor TrK_B_ gene expression, and the orexin 1 and 2 receptors, in rodent amygdala and/or hippocampus (Smith et al., 2014; Achua et al., submitted). Also BDNF, TrK_B_, AMPA receptor subunit GluA_1_ genes exhibit greater expression in trout dorsolateral pallium (Dl, homologous to hippocampus) (Carpenter et al., 2009b). Gene expression may also be predictive of the submissive (Stay) phenotype, in which SAM interaction plus FC stimulates elevated levels of cannabinoid 2 receptor (Cb_2_) in both dorsal and ventral hippocampus (Robertson et al., 2017). Interestingly, though both Stay and Escape phenotypes receive significant levels of aggression, neither the quantity nor the intensity of attack determines which behavioral phenotype is adopted (Figure 2), and therefore differences in hormone and gene expression in those groups are determined by the choice of behavioral response (Prince et al., 2015). Responses to this gradient are amenable to modification by anxiolytic treatments (such as exercise on the running wheel, familiarity with escape, NPS, the CRF_1_ antagonist antalarmin), which reverses highly anxious submissive behaviors and allows for escape (Carpenter et al., 2009b; Smith et al., 2016). Conversely, anxiogenic treatments (noradrenergic α_2A_ antagonist yohimbine, aggression) block escape behavior, and promote submission (Robertson et al., 2015; Smith et al., 2016).

While the overall general pattern of submission-, retreat or explore-, and aggression-related behaviors are virtually identical for Escape and Stay phenotypes (Figure 5), there are specific behaviors that identify the long-term phenotypic outcome on the first day of social interaction, before the eventual phenotype is obvious (Figure 6). Animals that Stay behave significantly more submissively than do those that eventually use the escape route, but both phenotypes display submission-related behaviors significantly more often than other behaviors. Retreat and exploring-related behaviors (including escape for that phenotype) are expressed significantly more than aggressive behaviors (they are smaller fish after all), and less than submissive behaviors in both phenotypes. It is well-known that subordinate fish, including trout, avoid the center of the water column, where dominant fish patrol (Winberg et al., 1991). A related suite of behaviors, including swimming at the bottom, swimming at the top, and sitting at the bottom are more frequently exhibited by trout that remain submissively (Stay) with the larger aggressor, even on the first day of social interaction prior to the final outcome. Trout that eventually escape do express these behaviors, just significantly less often than those that will become submissive (Figures 6C,D). This suggests that making the decision to adopt the submissive phenotype occurs very early, and that these fish may also be predisposed to be subordinate when paired with a size-matched conspecific. Trout that learn to escape (Carpenter and Summers, 2009) predominantly express different behaviors during their first social interaction, than those that remain submissively. Trout that eventually express the Escape phenotype are much more likely than non-escapers to turn toward their assailant (which is roughly three times larger) during an attack. Eventually escaping fish also spend significantly more movement directed at the escape route (hole) than submissive fish (Figures 6A,B). In fact, submissive fish virtually never do these two behaviors, which do occur, but so infrequently that these behaviors appear to be diagnostic for escaping trout. It remains to be demonstrated that fish of the Stay phenotype, which exhibit a physiologically conditioned response to a Pavlovian conditioned stimulus [after a week of pairing the CS with the large aggressor US (Carpenter and Summers, 2009)], also swim to the top or bottom in response to the CS alone.

In conclusion, the two behavioral phenotypes expressed by a population of rainbow trout exhibit early behavioral indicators of the development of those phenotypes. Surprisingly, aggression *per se*, does not seem to drive escape behavior; although, in contrast, trout that Stay receive slightly greater maximum attack intensity. This suggests that trout that don't escape are more stress vulnerable than those that do, which is borne out by previous work demonstrating that anxiolytic CRF_1_ antagonism allows submissive animals (trout and mice) to escape (Carpenter et al., 2009b; Robertson et al., 2015; Smith et al., 2016), and that CRF may promote indecision toward escape behavior (Figures 3C,D). Snap-shake, a behavior induced by icv CRF and blocked by CRF_1_ receptor antagonism, is performed primarily after escape-related behaviors by submissive fish, and quantity of snap-shake performance is positively correlated in escaping trout with the latency to escape. Although there are significant barriers to learn escape, some trout make their escapes while the larger aggressive fish is not observing their movements, and clearly time their approach and execution for that moment. The fish and rodent versions of the SAM demonstrate a simple decision-making paradigm that describes an intensity gradient of anxiety and depressive behaviors for animals as evolutionarily distant as rainbow trout and mice (Carpenter et al., 2007, 2009b; Carpenter and Summers, 2009; Smith et al., 2014, 2016; Prince et al., 2015; Robertson et al., 2015). This suggests that the phenotypic traits associated with staying submissively or escaping from socially aggressive interaction, are evolutionarily conserved across the vertebrate phyla, and are generated by similar neuroendocrine mechanisms.

Results from the new SAM conceptual model suggest that we can examine both susceptible and resilient individuals in real time, as well as begin to understand the behaviors and mechanisms that predict and produce these phenotypes. Behavioral outcomes in the SAM reflect changes in neurocircuitry and sensitivity to stressful sensory stimuli. While all individuals display submissive behavior during socially stressful interactions, susceptible individuals display specific types of submissive behavior, which are suggestive of withdrawal and behavioral inhibition. Resilient individuals on the other hand, face the social stress, learn from antagonistic social interactions and individuals, and use that information to escape from, and therefore ameliorate the socially stressful conditions. While increasing levels of anxiety result in a commensurate level of circumvention and indecision among resilient individuals, there appears to be a threshold level of anxiety that precludes resolving social stress through egress and commits an individual to submissive responses. While the data suggest interactive stress and arousal mechanisms (Carpenter et al., 2007, 2009b; Robertson et al., 2015; Smith et al., 2016) that produce adaptive behaviors for both escaping and submissive fish (Carpenter and Summers, 2009), which may be genetically and/or epigenetically heritable (Benus et al., 1991; Pottinger and Carrick, 1999; Øverli et al., 2002), and potentially reflect proactive and reactive stress-coping strategies (Koolhaas et al., 1999; Øverli et al., 2004, 2007), we also suspect that directional changes to the neural circuits and endocrine systems involved may also subvert decision-making (Carpenter and Summers, 2009; Carpenter et al., 2009b; Smith et al., 2014, 2016; Robertson et al., 2015) and result in anxious and depressive behaviors in all vertebrates, which may translate eventually to a broader understanding of psychological disorders (Riise et al., 2015; Keifer and Summers, 2016; Øverli and Sorensen, 2016; Vindas et al., 2016).

## Author contributions

Participated in all aspects of the experimental, analysis, and writing tasks including substantial contributions to conception and design as well as acquisition, analysis, and interpretation of data, drafting, and revising the written text, final approval of the version to be published, and accountability for all aspects of the work in ensuring that questions related to the accuracy or integrity of any part of the work are appropriately investigated and resolved: TRS, TLS, RC, and CS. Participated in substantial contributions to conception and design as well as acquisition, analysis, and interpretation of data, revising the written text, final approval of the version to be published, and accountability for all aspects of the work in ensuring that questions related to the accuracy or integrity of any part of the work are appropriately investigated and resolved: JS and DA. Participated in substantial contributions in design as well as acquisition, analysis, and interpretation of data, drafting and revising the written text, final approval of the version to be published, and accountability for all aspects of the work in ensuring that questions related to the accuracy or integrity of any part of the work are appropriately investigated and resolved: SY, BM, and TO.

### Conflict of interest statement

The authors declare that the research was conducted in the absence of any commercial or financial relationships that could be construed as a potential conflict of interest.
